# Education of pediatric subspecialty fellows in transport medicine: a national survey

**DOI:** 10.1186/s12887-017-0780-5

**Published:** 2017-01-13

**Authors:** Geoffrey E. Mickells, Denise M. Goodman, Ranna A. Rozenfeld

**Affiliations:** 1 Division of Critical Care Medicine, Ann & Robert H. Lurie Children’s Hospital of Chicago, Department of Pediatrics, Feinberg School of Medicine, Northwestern University, Chicago, IL USA; 2 Children’s Healthcare of Atlanta at Scottish Rite, Section of Pediatric Critical Care Medicine, Neonatology Associates of Atlanta, Atlanta, GA USA

**Keywords:** Fellow education, Transport medicine, Pediatric critical care medicine, Neonatal-Perinatal medicine, Pediatric emergency medicine

## Abstract

**Background:**

The transport of critically ill patients to children’s hospitals is essential to current practice. The AAP Section on Transport Medicine has raised concerns about future leadership in the field as trainees receive less exposure to transport medicine. This study identifies the priorities of pediatric subspecialty fellows, fellowship directors and nursing directors in transport medicine education.

**Methods:**

Internet based surveys were distributed to fellows, fellowship directors and nursing directors of transport teams affiliated with ACGME-approved fellowships in Neonatal-Perinatal Medicine (NPM), Pediatric Critical Care Medicine (PCCM), and Pediatric Emergency Medicine (PEM). Data collection occurred November 2013 to March 2014.

**Results:**

Four hundred and sixty-six responses were collected (357 fellows, 82 directors, 27 nursing directors): Six curricular elements were ranked by respondents: Transport Physiology (TP), Medical Control (MC), Vehicle Safety (VS), Medicolegal Issues (ML), Medical Protocols (MP) and State and Federal Regulations (SFR). Fellows and fellowship directors were not significantly different: TP (*p* = 0.63), VS (*p* = 0.45), SFR (*p* = 0.58), ML (*p* = 0.07), MP (*p* = 0.98), and MC (*p* = 0.36). Comparison of subspecialties found significant differences: PEM considered TP less important than NPM and PCCM (*p* < 0.001, *p* < 0.001), VS less important than NPM (*p* = 0.001). PEM viewed SFR and MC more important than PCCM (*p* = 0.006, *p* = 0.002); ML more important than PCCM and NPM (*p* = 0.001, *p* < 0.001). PCCM ranked MC more important than NPM (*p* = 0.004). Nursing directors considered TP less important than NPM and PCCM (*p* < 0.001, *p* = 0.002).

**Conclusions:**

When ranking curricular elements in transport medicine, fellows and fellowship directors do not differ, but comparison of subspecialties notes significant differences. A fellow curriculum in transport medicine will utilize these results.

**Electronic supplementary material:**

The online version of this article (doi:10.1186/s12887-017-0780-5) contains supplementary material, which is available to authorized users.

## Background

Transport of critically ill and injured infants and children is a crucial component of current pediatric practice. Transfers from other facilities are estimated to be 1.5% of visits of non-critically ill patients to pediatric emergency departments [1], from 0––100% of admissions to academic neonatal intensive care units depending on presence of obstetrical services [2], and up to one-third of admissions to pediatric intensive care units [3].

Previous Accreditation Council of Graduate Medical Education (ACGME) requirements for pediatric residents specify “participation in pre-hospital management and transport” as a component of training in treating acutely ill and injured children [4]. Previous studies demonstrate participation in Emergency Medical Services or pediatric transport teams occurs in just 60% of pediatric residencies, with variable team roles and experiences at those programs [5–7]. It is clear that pediatric residents do not receive uniform exposure to transport medicine. This is noted by the American Academy of Pediatrics Section on Transport Medicine (AAP SOTM) 2013 Consensus Statement on Interfacility Transport:

“Transport patient care remains an essential part of resident/fellow training especially in neonatology, pediatric critical care and pediatric emergency medicine, although the need for a physician presence on transport teams remains controversial. As resident/fellow training regulations and work hour restrictions have changed, fewer trainees are exposed to the unique learning environment of a transport program. The impact of this loss may become apparent as trainees advance to careers in critical care disciplines and transport medical direction” [8]

ACGME requirements for fellowship trainees in neonatal-perinatal medicine (NPM), pediatric critical care medicine (PCCM), and pediatric emergency medicine (PEM) are limited [9–11]. Similarly, content outlines for subspecialty board certification by the American Board of Pediatrics are sparse when addressing transport medicine [12–14]. The AAP SOTM recommends “fellowship programs in neonatology, pediatric critical care medicine and pediatric emergency medicine should include transport medicine and medical control training,” despite the limited requirements of other agencies [15].

There are no known studies evaluating how pediatric subspecialty fellows are taught transport medicine during fellowship, which concepts are considered most important in this education, or their preferred methods of learning. Further, the goals, priorities and expectations of other groups, including fellowship directors and nursing directors of transport teams, have not been explored.

With this study the investigators sought to prioritize the components of transport medicine most important to pediatric subspecialty fellows, pediatric subspecialty fellowship directors and nursing directors of neonatal and pediatric transport teams. Additionally, we aimed to describe the current state of education in transport medicine.

## Methods

This survey was designed as an online, prospective, cross sectional survey of three different groups of stakeholders for neonatal and pediatric transport medicine: nursing directors of transport teams affiliated with academic medical centers, fellows in pediatric subspecialties (Neonatal-Perinatal Medicine, Pediatric Critical Care Medicine, Pediatric Emergency Medicine) and fellowship directors in these same subspecialties. Included subjects were fellows in accredited fellowship programs in one of the three subspecialties during the 2013–2014 academic year, fellowship directors of these fellowship programs and nursing directors of pediatric transport teams affiliated with hospitals that had at least one accredited fellowship program in the subspecialties of interest. Participants in non-accredited fellowships or transport teams not affiliated with a fellowship program were excluded. Fellowship Directors were chosen as a survey population as they are directly responsible for the curriculum decisions in their programs. Nursing directors were included instead of transport team medical directors, who are typically physicians, in order to provide multidisciplinary input and because nursing directors are more likely to interact with subspecialty fellows in the course of patient transports.

To accomplish the primary aim of the study, a survey was conducted with a forced ranking of six elements of transport medicine for a proposed curriculum, transport physiology (TP), vehicle safety (VS), State and Federal Regulations (SFR), medicolegal issues (ML), medical protocols (MP) and principles of medical control (MC). These elements were chosen after a review of available literature and discussion with individuals with expertise in transport medicine [15, 16]. This included fellows, fellowship directors and the nursing director of the transport team at our institution and further discussion with members of the AAP SOTM. Descriptions of current curricula in transport medicine for fellows, including content and teaching methods, were obtained to measure the secondary aims.

Surveys were developed with input from individuals in each category of stakeholder to improve content validity. This included four fellows, one fellowship director and the nursing director of the transport team from our institution. A sociologist assisted in survey design to help limit bias, address question validity and improve overall survey tool reliability by suggesting techniques for phrasing and formatting of questions online and the associated benefits and limitations of various alternatives. Critical appraisal of questions was provided. Strategies for analytical methods to compare answers from respondents from a single institution were discussed.

Surveys for fellows included no more than 20 items on 15 pages, fellowship directors’ survey included no more than 19 items on 15 pages, and nursing directors’ no more than 13 questions on 10 pages (Additional file 1). Questions were not randomized and adaptive questioning was utilized based on participant responses resulting in variable number of questions in each survey. All questions presented to a respondent required an answer. Participants were able to review their responses through the use of navigation buttons within the survey. IP addresses, collected by default by SurveyMonkey.com, were examined to prevent multiple entries from a single respondent. It was decided a priori to include incomplete surveys in the final analysis.

The study met criteria for exemption from full review by the Institutional Review Board of Ann & Robert H. Lurie Children’s Hospital of Chicago by fulfilling 45 CFR 46.101 (b) (1).

Informed consent was obtained through disclosure in the front matter of the survey, which included description of the aims of the study, the expected length of time to complete the survey, description of potential harms and benefits, and methods of data collection. Participation in the survey was considered indicative of informed consent. Identifying data collected in the survey consisted of hospital affiliation, subspecialty and in the case of fellows, year of fellowship. The hospital affiliation was coded immediately through random number generation and the coding key was stored under standard password protection on computers only available to the investigators. Data supporting the conclusions of this study are not publicly available and will not be shared as it contains information that would compromise participant privacy and consent.

This was a closed survey, with participants recruited through direct email communication by the investigators. Fellowship directors and support staff coordinators were identified through listings on the Fellowship and Residency Electronic Interactive Database maintained by the American Medical Association. Email addresses were confirmed, where possible, by evaluation of individual fellowship websites. To reach fellowship trainees, the investigators emailed distinct invitations to the fellowship directors and fellowship coordinators, asking for assistance in forwarding the invitations to fellows within their programs. Nursing directors were identified from the AAP SOTM database, through the AAP SOTM listserv, the Commission on Accreditation of Medical Transport Systems Database and through direct phone calls to transport teams. Invited participants were directed to the appropriate survey for their position via a dedicated SurveyMonkey.com web link for each survey tool. The initial invitation to participate was sent in November 2013, with follow-up reminders sent monthly through April 2014. No incentives for participation were provided.

Publicly available counts of fellows are not considered reliable due to positions being offered outside of the Match and fellows who leave fellowship before completion. Additionally, it was not possible to identify the number of unique site visitors, or how many individuals started the survey then exited prior to answering any questions through available means on the SurveyMonkey.com website. Without this information, neither the view rate nor the participation rate could be calculated.

Descriptive statistics were calculated and compared for statistical significance utilizing Wilcoxon Rank Sum, Kruskal-Wallis and Chi-Square tests. Statistical significance was defined as *p-*values ≤0.05. When multiple pairwise comparisons were done, the Bonferroni correction was used. All comparisons were made utilizing SAS, version 9.4 (SAS Institute, Cary, NC). Results are presented adhering to the Checklist for Reporting Results of Internet E-Surveys (CHERRIES) [17, 18].

## Results

There were a total of 466 responses, 357 from fellows, 82 from fellowship directors and 27 from nursing directors. Response rates for fellows were estimated due to limitations in data from the National Residency Matching Program Specialties Matching Service, with an estimated response rate for fellows in Neonatal-Perinatal Medicine of 19%, Pediatric Critical Care Medicine of 25%, and Pediatric Emergency Medicine of 30%. For fellowship directors, the response rates were 40% for Neonatal-Perinatal Medicine, 42% for Pediatric Critical Care Medicine, and 23% for Pediatric Emergency Medicine. The nursing director response rate was 35%. A total of 90 programs had at least one respondent. The completion rate (indicating complete replies with no unanswered questions) for the survey was 100% for nursing directors and fellowship program directors, and 91.6% for fellows.

**Fig. 1:**
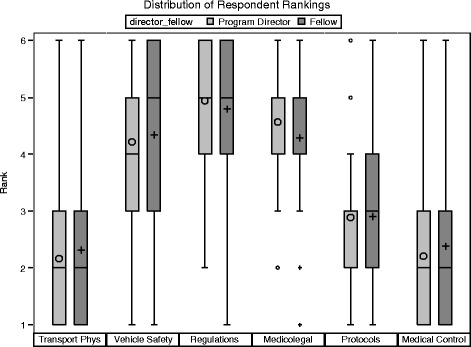
Distribution of Respondent Rankings by Physician Type. Comparison of program directors and fellows rankings. *Box plots* represent interquartile range with symbols inside the *box plot* representing the mean score. Symbols outside the *box plot* represent outliers

The primary outcome of the forced ranking from most important to least of six curricular elements found that fellows and fellowship directors were not significantly different across curricular elements. Transport Physiology (TP) (Z-score: −0.52, *p* = 0.63, Wilcoxon Rank-sum test), Vehicle Safety (VS) (−0.76, *p* = 0.45), State & Federal Regulations (SFR) (0.56, *p* = 0.58), Medicolegal Issues (ML) (1.82, *p* = 0.07), Medical Protocols (MP) (0.03, *p* = 0.98), and Medical Control (MC) (−0.91, *p* = 0.36) (Fig. 1).

**Fig. 2:**
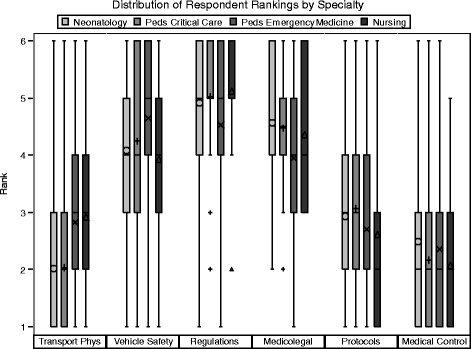
Distribution of Respondent Rankings by Physician Specialty. Comparison of specialty rankings. *Box plots* represent interquartile range with symbols inside the *box plot* representing the mean score. Symbols outside the *box plot* represent outliers

When respondents were grouped by subspecialties significant differences arose regarding importance of specific curricular elements (Fig. 2). Pediatric Emergency Medicine subspecialty physicians (fellows and fellowship directors) considered Transport Physiology less important than physicians in Neonatal-Perinatal Medicine and Pediatric Critical Care Medicine (*p* < 0.001 and *p* < 0.001), and Vehicle Safety less important than Neonatal-Perinatal Medicine providers (*p* = 0.001). Conversely, Pediatric Emergency Medicine physicians viewed State & Federal Regulations and Medical Control more important than Pediatric Critical Care Medicine physicians (*p* = 0.006 and *p* = 0.002 respectively); and Medicolegal Issues higher than physicians in both Pediatric Critical Care Medicine and Neonatal-Perinatal Medicine (*p* = 0.001 and <0.001 respectively). Pediatric Critical Care Medicine providers placed greater importance on Medical Control than Neonatal-Perinatal Medicine physicians (*p* = 0.004).

Nursing directors considered Transport Physiology less important than physicians in Neonatal-Perinatal Medicine and Pediatric Critical Care Medicine (*p* < 0.001 and *p* = 0.002). They did not differ from physicians in any other element.

Fifty one percent of fellows reported having experience in transport medicine prior to starting fellowship with experiences ranging from ambulance ride-alongs while a medical student to serving as attending physician on patient transports in the time between residency and fellowship. Other fellows reported that their experience with transport was limited to taking phone calls from referring medical facilities, with no in-person participation with patient transfers during residency. Despite this variable experience, groups dichotomized into experienced or not experienced did not differ statistically except in State & Federal Regulations (Z-Score −2.58, *p* = 0.007, Wilcoxon Rank-Sum Test) which was considered less important by those with experience.

The ACGME requires minimum patient volumes in order to provide adequate education for pediatric subspecialty fellows. For example, Pediatric Critical Care Medicine fellowships must have a minimum of 700 annual admissions to the Pediatric Intensive Care Unit (PICU). Due to location and specialty services available, children’s hospitals have wide differences in patient volume: annual admissions for responding Neonatal-Perinatal Medicine fellowship programs ranged from 335 to 2000 with a median of 907; for PICU respondents the range was 700–3000, with a median of 1500; and for Pediatric Emergency Medicine respondents, annual visits ranged from 27,000 to 125,000 patients with a median of 58,000. Transport teams report patient retrievals ranging from 123 to 4940 annual transports with a median of 1115. When dichotomized into large and small programs by specialty using the median, there were no differences between any groups except that Pediatric Critical Care Medicine providers at small programs (<1500 admissions) ranked State & Federal Regulations more important than Pediatric Critical Care Medicine practitioners at larger programs (Z-Score −2.62, *p* = 0.009, Wilcoxon Rank Sum Test). All other comparisons for each curricular element were not statistically significant.

94.1% of physicians considered fellow education in transport medicine important or very important. This included 90% of fellowship directors, however only 55% of program directors reported having a formal curriculum in transport medicine for their fellows. Meanwhile, only 39.1% of fellows reported receiving formalized education in transport, while 38.2% stated no curriculum existed, and 22.6% were unsure. This difference between fellows and directors was significant, with directors over twice as likely to report a curriculum present (Chi-Square, *p* = 0.001, OR = 2.24, 95% CI: 1.37–3.67).

Methods to teach transport medicine included lectures, assigned readings and one-on-one instruction with an attending physician. According to program directors, lectures and one-on-one discussions with faculty were the most frequently used methods of instruction for each element, a finding echoed by the fellows (Table 1). Fellows were much more likely to report an element as not being formally taught, which was statistically significant for all elements (Chi-Square, TP 56.91, *p* < 0.001, VS 12.28, *p* < 0.001, SFR 11.94, *p* < 0.001, ML 44.99, *p* < 0.001, MP 61.79, *p* < 0.001, MC 129.66, *p* < 0.001). Differences in reporting existed between fellows and program directors regarding the use of one-on-one discussions as part of their curriculum for some elements. Program directors said they were used but fellows did not at significantly different rates for State & Federal Regulations (Chi-Square, 10.39, *p* = 0.001), Medicolegal Issues (12.67, *p* < 0.001), Medical Protocols (5.46, *p* = 0.02) and Medical Control (9.67, *p* = 0.019).

**Table 1:** Program directors’ and fellows’ report of teaching methods

	Lectures		Computer Learning		Assigned Readings		1 on 1 Discussion		Other		Element Not Taught	
	PD	Fellows	PD	Fellows	PD	Fellows	PD	Fellows	PD	Fellows	PD	Fellows
TP	60%	28%^*^	3%	4%	14%	8%	41%	31%	10%	8%	16%	62%^*^
VS	29%	24%	3%	5%	5%	5%	23%	17%	20%	14%	34%	55%^*^
SFR	21%	25%	6%	6%	8%	6%	31%	16%^*^	15%	10%	34%	54%^*^
ML	34%	31%	3%	6%	9%	5%	45%	25%^*^	15%	8%	23%	63%^*^
MP	35%	31%	6%	5%	16%	8%^*^	45%	32%^*^	24%	14%	20%	68%^*^
MC	38%	33%	5%	5%	10%	5%	65%	46%^*^	21%	13%	10%	77%^*^

*PD* Program Director, *TP* Transport Physiology, *VS* Vehicle Safety, *SFR* State & Federal Regulations, *ML* Medicolegal Issues, *MP* Medical Protocols, *MC* Medical Control

% of respondents reporting an element was taught using a particular teaching method

^*^ = statistically significant differences between fellows and program director reported rates

When asked their preferred learning methods, fellows selected lectures (69% of respondents) and one-on-one discussions with faculty members (60%) most frequently, with assigned readings (29%) and computer modules (44%) less popular. Ten percent of respondents chose other methods, with experiential learning either through simulation or active participation in patient transports frequently suggested in the free text responses.

## Discussion

This survey is the first investigation that attempts to identify and prioritize the components of a transport curriculum to best address the composite needs of fellowship trainees, fellowship directors and transport nursing directors. Given the variable exposure to transport medicine in residency and the common interaction with transported patients in daily clinical care, fellowship stands as the only opportunity to develop skills related to transport medicine in a supervised environment. Therefore, to address the concerns raised by the AAP SOTM, it is vital to understand the ways transport medicine is presently taught, what elements should have priority, and how to deliver such information.

Our study confirms findings from previous studies regarding variable exposure to transport medicine for pediatric residents [5–7]. This study shows that there is broad agreement that Transport Physiology, Principles of Medical Control and Medical Protocols are of higher priority than Vehicle Safety, State and Federal Regulations or Medicolegal Issues. However, when subspecialty groups and nursing are compared, differences in the relative importance of these elements do appear. Most notable are the differences between Pediatric Emergency Medicine physicians who ranked Medical Protocols higher and Transport Physiology as less important than did neonatologists or intensivists. Also of interest is the greater importance assigned to Principles of Medical Control by intensivists compared to neonatologists. Both these differences may exist due to variances in training, experience or the philosophy of care that exist between the three disciplines. For example, one could foresee that Pediatric Emergency Medicine physicians place higher priority on Medical Protocols because of frequent encounters with Emergency Medical Services providers who typically function in the field with offline medical control provided by detailed protocolized order sets [19]. Meanwhile Pediatric Critical Care Medicine physicians most frequently provide medical control using frequent, direct contact with transport team members.

The discrepancy among directors between the perceived importance for transport medicine education for fellows, but concurrently less evidence of a formal curriculum, is interesting. It may suggest that program directors have overstated their feelings towards transport medicine or are unsure of what a formal curriculum in transport medicine should include. It is possible that fellowship directors would readily incorporate a ready-to-use curriculum were it to be provided.

These findings may inform development of a curriculum in transport medicine for pediatric subspecialty fellows. Any such attempt should consider the diverse needs found in our study. While it is the investigators’ belief that all the elements deserve discussion, it is clear that equal time for each element is not desired by stakeholders. This suggests that some flexibility is needed within a finished curriculum to allow fellowship directors or fellows to address their self-identified needs.

The manner in which transport medicine is taught is also a consideration. While lectures were the most preferred choice for learning transport medicine, principles of adult learning suggest that these are likely to be less effective than self-directed methods of learning, which would be more efficacious. As adults have a tendency to be more practical and problem based in their learning styles [20], it perhaps is not surprising that one-on-one discussions with attending physicians, assumed to be about points relevant to specific patient transports, were also preferred by fellows. The difficulty in curriculum development is to provide a standardized foundation, and monitoring informal discussions for content and validity is impossible. Immersive experiences for fellows through required rotations with the transport team were a frequent suggestion, but this approach suffers from a similar lack of content control when applied broadly. While this does not negate the value of first-hand experience in understanding the austere transport environment, it suggests that more controlled experiences, such as high-fidelity simulation, have a place in any proposed curriculum.

This study has several limitations. The most important is the relatively low response rate. Traditionally, response rates of >60% are expected for survey research when a study population receives a survey via phone call or mail. However, adequate response rates for internet-based surveys is less certain and lacks consensus. Multiple studies demonstrate that email based surveys have lower response rates than surveys sent by mail. The Checklist for Reporting Results of Internet E-Surveys (CHERRIES), analogous to the CONSORT or QUORUM statements for randomized controlled trials and systematic reviews, suggests that response rates of less than 40% in general internet surveys may still have significant validity [17]. The response rate to our study is similar to those of another recent email based survey of physicians, in particular given that we did not offer monetary incentives for completion [21]. Noting that 94% of respondents considered transport medicine important for pediatric subspecialty fellows, the possibility of a strong selection bias is high. If only surveying those who believe in the utility of transport medicine education, future proposed curricula may not meet the needs of all subspecialty fellows.

The limited response rate also prevented further analysis that would have corroborated data for individuals affiliated with the same institution. Were this possible, the higher internal validity may have yielded further insights, particularly for discussing the discrepancy between content delivery reported by fellowship directors and the responses from fellows that suggest they are unaware of said teaching.

Limiting the population of our study was necessary for convenience, but additional insights may have been reached had we surveyed attending physicians in these fields regarding their experiences and needs when providing medical control for the transport of critically ill or injured children and neonates.

Finally, because the field of transport medicine is an underdeveloped field of study with highly variable pediatric residency training experiences we elected to design the survey to address investigator-selected curricular elements. Other methods of completing needs assessments, such as Delphi methodology, assume that respondents have a degree of prior experience and expertise in the field of study to identify needs beyond those suggested by the investigators. We did not feel that we could reliably expect pediatric subspecialty fellows to have this degree of familiarity with transport medicine based on prior research. Potentially in the future, after completion of a curriculum in transport medicine based on the curricular elements assessed in our study, a more comprehensive needs assessment could be completed. Because of these limitations, this study does not represent a comprehensive needs assessment.

## Conclusions

The transport of critically ill and injured infants and children is a fundamental and daily component of practice for pediatric subspecialists. Despite this, trainees in pediatrics and associated subspecialty fields receive an uneven exposure and education in transport medicine. Our study finds notable differences between subspecialty groups, but not between fellowship directors and fellows within each field, regarding relative importance of elements in transport medicine. The study also demonstrates a discrepancy between fellowship directors and trainees regarding current delivery and receipt of educational content in transport medicine. The results of this study will inform decision making regarding content and educational methods in the creation of a comprehensive curriculum in transport medicine for pediatric subspecialty fellows.

## Additional file

Additional file 1: Survey Tools. (DOCX 37 kb)

## Abbreviations

AAP: American Academy of Pediatrics
ACGME: Accreditation Council of Graduate Medical Education
MC: Principles of medical control
ML: Medicolegal issues
MP: Medical protocols
NPM: Neonatal-Perinatal Medicine
PCCM: Pediatric Critical Care Medicine
PEM: Pediatric Emergency Medicine
PICU: Pediatric Intensive Care Unit
SFR: State and Federal Regulations
SOTM: Section on Transport Medicine
TP: Transport Physiology
VS: Vehicle Safety
