# Supplementary material for: Education of pediatric subspecialty fellows in transport medicine: a national survey
Source: BMC Pediatr. 2017 Jan 13;17:13. doi: 10.1186/s12887-017-0780-5 (PMC5237255; doi:10.1186/s12887-017-0780-5)
Supplement: Additional file 1: — Survey Tools. (DOCX 37 kb) [file 12887_2017_780_MOESM1_ESM.docx]

APPENDIX A:

Part 1: FELLOWS SURVEY

I would like to start by asking your opinions on transport medicine. For the survey, the following definitions will apply (pop up box)

The American Academy of Pediatrics Section on Transport Medicine has published guidelines for the transport of neonatal and pediatric patients that are regularly updated. High priority elements of these guidelines are defined as follows:

- Transport physiology includes concepts such as the physical gas laws and changes associated with increased altitude, as well as noise, vibration and barometric stresses of the transport environment (fixed wing or rotor wing aircraft, ground ambulances)
- Vehicle Safety includes concepts such as appropriate use of lights and sirens, risks associated with various transport modalities, specific safety equipment unique to the transport environment and management of these potential risks.
- State and Federal Regulations includes concepts such as certification of transport teams and facilities (i.e. helipads), Federal Aviation Administration rules regarding medical equipment in flight, and rules governing the number of hours pilots may work.
- Medicolegal Issues includes concepts such as EMTALA, liability considerations relating to transfer of care from one institution, HIPAA regulations, and informed consent
- Medical Protocols includes concepts such as prewritten treatment plans, including medications and doses, to be followed by nurses, respiratory therapists or EMS personnel with limited input from a physician.
- Medical Control includes concepts such as triage, information gathering from the referring hospital, methods of giving advice to other providers who may have limited experience with caring for critically ill or injured children, interactions with transport team personnel via various means and documentation.

1. Please rank the following concepts (1=most important, 6=least important) that you believe are most important for pediatric critical care fellows to learn in order to be best served as medical control physicians for patient transports:

| Concept | Rank |
| --- | --- |
| Transport Physiology |  |
| Vehicle Safety |  |
| State and Federal Regulation |  |
| Medicolegal Issues |  |
| Medical Protocols |  |
| Medical Control |  |

1. How important do you consider transport medicine to be in your education as a pediatric critical care fellow?
   1. Very important
   2. Somewhat important
   3. Neither important or unimportant
   4. Somewhat unimportant
   5. Very unimportant
2. Describe your overall level of comfort in managing the transfer of a critically ill or injured child from an outside facility to your PICU as the medical control physician:
   1. Expert
   2. Proficient
   3. Competent
   4. Advanced Beginner
   5. Novice
3. Prior to fellowship, did you have any prior experience with transport medicine (such as taking phone calls from referring hospitals, participating in patient retrievals or ambulance ride-alongs, or in another non-resident role such as working as an EMT/Paramedic)?
   1. Yes (please describe your experience)
   2. No

At this time I would like to learn more about your fellowship program:

1. Please name your current fellowship program (this information will remain confidential and will not be published):
2. What is your current year of critical care fellowship
   1. 1^st^
   2. 2^nd^
   3. 3^rd^
   4. Fellow in a combined specialty program (e.g. cardiology and critical care)
3. How often do the critical care fellows (all fellows combined) at your current institution answer phone calls from referring hospitals for advice, stabilization and treatment recommendations for children?
   1. More than once a day
   2. Less than once a day, but more than once a week
   3. Less than once a week
   4. Less than once a month
   5. Never
4. Does your current institution have a dedicated critical care transport team for children (not neonates)?
   1. Yes
   2. No
   3. Unsure
5. If yes to question 8, do you as a critical care fellow provide medical control (including but not limited to triage decisions, medical decision making) for the transport team during patient retrievals from referring hospitals?
   1. Yes
   2. No
6. If no to question 9, who provides medical control for the transport team when they transport pediatric patients?
   1. Emergency Department physicians
   2. Team follows protocols
   3. Other
   4. Unsure
7. Do you as a critical care fellow at your current institution provide medical control for non-affiliated transport teams (commercial entities, transport teams from other institutions, or community EMS crews)?
   1. Yes
   2. No
   3. Unsure
8. Is it typical for the pediatric transport team at your current institution to include a physician (attending, fellow, pediatric or emergency medicine resident) during patient retrievals?
   1. Physicians are ALWAYS part of our team composition for pediatric patients
   2. Physicians are SOMETIMES a part of our team composition
   3. Physicians are not part of our team composition

I would now like to learn about what sort of education in transport medicine you receive during your fellowship:

1. Does your fellowship program require fellows to participate in patient retrievals with transport teams?
   1. Yes
   2. No
   3. Physicians do not accompany our transport team on patient retrievals
2. If yes to question 12, what are the requirements for fellows? (select all that apply)
   1. Required rotation
   2. Required part of scheduled call duties
   3. Other (please explain)
3. If yes to question 12, is there a minimum number of patient retrievals fellows are required to complete during fellowship?
   1. Yes, more than 10
   2. Yes, between 5 and 10
   3. Yes, less than five
   4. No minimum requirement
   5. Unsure
4. Are fellows allowed to participate in patient transports (e.g. as optional moonlighting)?
   1. Yes
   2. No
5. Does your fellowship have a curriculum to teach concepts in transport medicine to fellows?
   1. Yes
   2. No
   3. Unsure
6. Describe the methods by which your fellowship has taught these concepts in transport medicine: Select all that apply

|  | Lectures | Computer Modules | Assigned Readings | One on one feedback from attending | Other (Please explain) | Concept not taught |
| --- | --- | --- | --- | --- | --- | --- |
| Transport Physiology |  |  |  |  |  |  |
| Vehicle Safety |  |  |  |  |  |  |
| State and Federal Regulations |  |  |  |  |  |  |
| Medicolegal Issues |  |  |  |  |  |  |
| Medical Protocols |  |  |  |  |  |  |
| Medical Control |  |  |  |  |  |  |

1. Describe your knowledge level for each of these concepts in transport medicine:

|  | Novice | Advanced Beginner | Competent | Proficient | Expert |
| --- | --- | --- | --- | --- | --- |
| Transport Physiology |  |  |  |  |  |
| Vehicle Safety |  |  |  |  |  |
| State and Federal Regulations |  |  |  |  |  |
| Medicolegal Issues |  |  |  |  |  |
| Medical Protocols |  |  |  |  |  |
| Medical Control |  |  |  |  |  |

Part 2: FELLOWSHIP DIRECTORS SURVEY

I would like to start by asking your opinions on transport medicine and the way in which you teach fellows. For the following survey, the following definitions will apply (pop up box)

The American Academy of Pediatrics Section on Transport Medicine has published guidelines for the transport of neonatal and pediatric patients that are regularly updated. High priority elements of these guidelines are defined as follows:

- Transport physiology includes concepts such as the physical gas laws and changes associated with increased altitude, as well as noise, vibration and barometric stresses of the transport environment (fixed wing or rotor wing aircraft, ground ambulances)
- Vehicle Safety includes concepts such as appropriate use of lights and sirens, risks associated with various transport modalities, specific safety equipment unique to the transport environment and management of these potential risks.
- State and Federal Regulations includes concepts such as certification of transport teams and facilities (i.e. helipads), Federal Aviation Administration rules regarding medical equipment in flight, and rules governing the number of hours pilots may work.
- Medicolegal Issues includes concepts such as EMTALA, liability considerations relating to transfer of care from one institution, HIPAA regulations, and informed consent
- Medical Protocols includes concepts such as prewritten treatment plans, including medications and doses, to be followed by nurses, respiratory therapists or EMS personnel with limited input from a physician.
- Medical Control includes concepts such as triage, information gathering from the referring hospital, methods of giving advice to other providers who may have limited experience with caring for critically ill or injured children, interactions with transport team personnel via various means and documentation.

1. Please rank the following concepts (1=most important, 6=least important) that you believe are most important for pediatric critical care fellows to learn in order to be best served as medical control physicians for patient transports:

| Concept | Rank |
| --- | --- |
| Transport Physiology |  |
| Vehicle Safety |  |
| State and Federal Regulation |  |
| Medicolegal Issues |  |
| Medical Protocols |  |
| Medical Control |  |

1. How important do you consider transport medicine to be in the education of pediatric critical care fellows?
   1. Very important
   2. Somewhat important
   3. Neither important or unimportant
   4. Somewhat unimportant
   5. Very unimportant
2. Does your fellowship have a curriculum to teach concepts in transport medicine to fellows?
   1. Yes
   2. No
   3. Unsure
3. Describe the methods by which your fellowship has taught these concepts in transport medicine: (Check all that apply)

|  | Lectures | Computer Modules | Assigned Readings | One on one feedback from attending | Other (Please explain) | Concept not taught |
| --- | --- | --- | --- | --- | --- | --- |
| Transport Physiology |  |  |  |  |  |  |
| Vehicle Safety |  |  |  |  |  |  |
| State and Federal Regulations |  |  |  |  |  |  |
| Medicolegal Issues |  |  |  |  |  |  |
| Medical Protocols |  |  |  |  |  |  |
| Medical Control |  |  |  |  |  |  |

1. Does your fellowship program require fellows to participate in patient retrievals with transport teams?
   1. Yes
   2. No
   3. Physicians do not accompany our transport team on patient retrievals
2. If yes to question 5, what are the requirements for fellows? (select all that apply)
   1. Required rotation
   2. Required part of scheduled call duties
   3. Other (please explain)
3. If yes to question 5, is there a minimum number of patient retrievals fellows are required to complete?
   1. Yes, more than 10
   2. Yes, between 5 and 10
   3. Yes, less than five
   4. No minimum requirement
   5. Unsure
4. Are fellows allowed to participate in patient transports (e.g. as optional moonlighting)?
   1. Yes
   2. No

I would now like to learn more about your fellowship program and your PICU:

1. Please name your current fellowship program (this information will remain confidential and will not be published):
2. Please quantify the number of fellows in your program for the 2013-2014 Academic Year:
   1. First year fellows ___
   2. Second year fellows ___
   3. Third year fellows ___
   4. Fellows in combined programs (e.g. Cardiology and Critical Care) ___
3. As of the 2013-2014 academic year, please quantify the total current number of beds in your institution’s PICU (do not include dedicated cardiac ICU beds):
   1. Does your institution have a physically separate Cardiac ICU?
      1. How many beds does it contain?
4. How many admissions did your PICU have in 2012 calendar year?
5. Does your institution have a dedicated critical care transport team for children (not neonates)?
   1. Yes
   2. No
   3. Unsure
6. Do critical care fellows at your institution provide medical control (including but not limited to triage decisions, medical decision making or disposition within the hospital) for the transport team during patient retrievals from referring hospitals?
   1. Yes
   2. No
7. Do critical care fellows at your institution provide medical control for non-affiliated transport teams (commercial entities, transport teams from other institutions, or community EMS crews)?
   1. Yes
   2. No
   3. Unsure
8. How often do the critical care fellows (all fellows combined) at your institution answer phone calls from referring hospitals – either directly or through a transport team - for advice, stabilization and treatment recommendations for children?
   1. More than once a day
   2. Less than once a day, but more than once a week
   3. Less than once a week
   4. Less than once a month
   5. Never
9. Is it typical for the pediatric transport team at your current institution to include a physician (attending, fellow, pediatric or emergency medicine resident) during patient retrievals?
   1. Physicians are ALWAYS part of our team composition for pediatric patients
   2. Physicians are SOMETIMES a part of our team composition
   3. Physicians are not part of our team composition

Part 3: NURSING DIRECTOR SURVEY

1. Please name your current affiliated pediatric hospital (this will remain confidential and will not be published):
2. How many pediatric patient transports were completed in 2012 by your transport team (do not include neonates):
3. List percentage of retrievals completed by each transport mode (must = 100%)

| Ground Ambulance |  |
| --- | --- |
| Rotor-wing |  |
| Fixed wing |  |

1. Is your transport team a dedicated team (e.g. staff members do not have primary bedside patient responsibilities while on a shift with the transport team)?
   1. Yes
   2. No
   3. Unsure
2. Is it typical for the pediatric transport team at your current institution to include a physician (attending, fellow, pediatric or emergency medicine resident) during patient retrievals?
   1. Physicians are ALWAYS part of our team composition for pediatric patients
   2. Physicians are SOMETIMES a part of our team composition
   3. Physicians are not part of our team composition
3. What is the typical composition of your team for pediatric critical care transports?
   1. Number of Physicians
   2. Number of Advanced Practice Nurses
   3. Number of Registered Nurses
   4. Number of Respiratory Therapists
   5. Number of Paramedics/EMT personnel
4. Do pediatric critical care fellows at your current institution provide medical control (including but not limited to triage decisions or medical decision making) for the transport team during pediatric patient retrievals from referring hospitals?
   1. Yes
   2. No
5. If no to question 7, who provides medical control to the transport team during patient retrievals?
   1. Free response

[Following info will be in pop-up box]

*The American Academy of Pediatrics Section on Transport Medicine (AAP, Woodward et al 2007) has published guidelines for the transport of neonatal and pediatric patients that are regularly updated. The areas addressed include:*

- *Transport physiology includes concepts such as the physical gas laws and changes associated with increased altitude, as well as noise, vibration and barometric stresses of the transport environment (fixed wing or rotor wing aircraft, ground ambulances)*
- *Vehicle Safety includes concepts such as appropriate use of lights and sirens, risks associated with various transport modalities, specific safety equipment unique to the transport environment and management of these potential risks.*
- *State and Federal Regulations includes concepts such as certification of transport teams and facilities (i.e. helipads), Federal Aviation Administration rules regarding medical equipment in flight, and rules governing the number of hours pilots may work.*
- *Medicolegal Issues includes concepts such as EMTALA, liability considerations relating to transfer of care from one institution, HIPAA regulations, and informed consent*
- *Medical Protocols includes concepts such as prewritten treatment plans, including medications and doses, to be followed by nurses, respiratory therapists or EMS personnel with limited input from a physician.*
- *Medical Control includes concepts such as triage, information gathering from the referring hospital, methods of giving advice to other providers who may have limited experience with caring for critically ill or injured children, interactions with transport team personnel via various means and documentation.*

1. Please rank the following concepts (1=most important, 6=least important) that you believe are most important for pediatric critical care fellows to learn in order to be best served as medical control physicians for patient transports

| Concept | Rank |
| --- | --- |
| Transport Physiology |  |
| Vehicle Safety |  |
| State and Federal Regulations |  |
| Medicolegal Issues |  |
| Medical Protocols |  |
| Medical Control |  |
